# Tin Oxide: The Next Benchmark Transport Material for Organic Solar Cells?

**DOI:** 10.1021/acsenergylett.4c02285

**Published:** 2025-02-20

**Authors:** David Garcia Romero, Lorenzo Di Mario, Maria Antonietta Loi

**Affiliations:** Photophysics and OptoElectronics, Zernike Institute for Advanced Materials, University of Groningen, Nijenborgh 3, Groningen, 9747 AG, The Netherlands

## Abstract

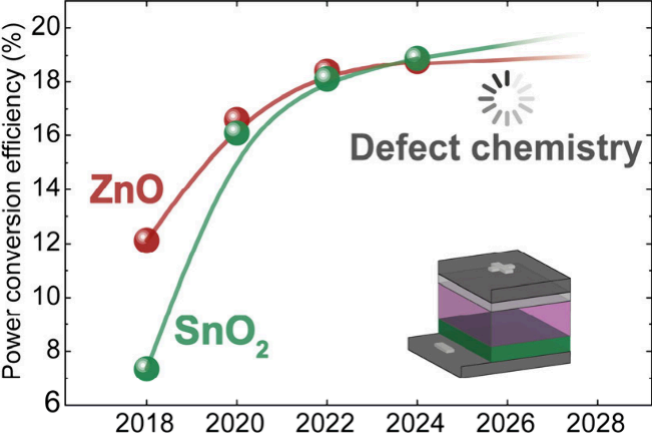

Organic solar cells (OSCs) are one of the most promising emerging photovoltaic technologies due to the rapid increase in efficiency in recent years. While efficiencies over 20% have been reported in laboratory scale devices using the conventional (p-i-n) structure, OSCs with inverted (n-i-p) structures still underperform, reaching values around 18%. Tin oxide (SnO_2_) has recently emerged as a promising transport layer for OSCs. Yet, some reproducibility challenges shown by the literature have hindered the full adaptation of this electron transport layer (ETL) by the organic solar cell community. This Perspective evaluates the current status of investigation for SnO_2_ as the transport layer for OSCs, focusing on its integration into state-of-the-art systems and highlighting the challenges toward its implementation. We examine which strategies lead to the most efficient and stable devices using SnO_2_ and give a critical view of whether this material can soon become the next benchmark electron transport layer for OSCs.

Organic solar cells (OSCs) have experienced a renaissance in the past years driven by the development of non-fullerene acceptors, which has led to unforeseeable power conversion efficiencies (PCE) of just above 20%.^1,2^ While this success is mainly determined by the synthesis of polymers and small molecules with improved charge generation, separation, and transport, the development of suitable electron and hole transport layers has also played a distinctive role in such progress.^3^ The arrangement of these transport layers, in either the conventional (or p-i-n) or the inverted (or n-i-p) configurations, significantly impacts the solar cell performance. In particular, OSCs with an inverted structure are considered more suitable for commercialization, thanks to the higher device stability and compatibility with scale-up manufacturing.^4,5^ However, the evolution of the record efficiencies for single-junction OSCs shows that despite the PCE having increased consistently over time for both structures, the conventional has overperformed the inverted one (Figure 1). Such an efficiency gap is determined mainly by the lack of transport layers for the inverted structure, with as high carrier selectivity and ideal energy-level alignment as the ones used in the conventional ones.^3^ Moreover, in some cases, spontaneous vertical phase segregation occurs inside the active layer,^6^ with the p-type polymer segregating toward the substrate, has been shown to favor the conventional configuration.^7,8^ Besides these phenomena, which do not appear to be universal, there are no fundamental reasons why the n-i-p structure should be worse than the p-i-n.

**Figure 1:**
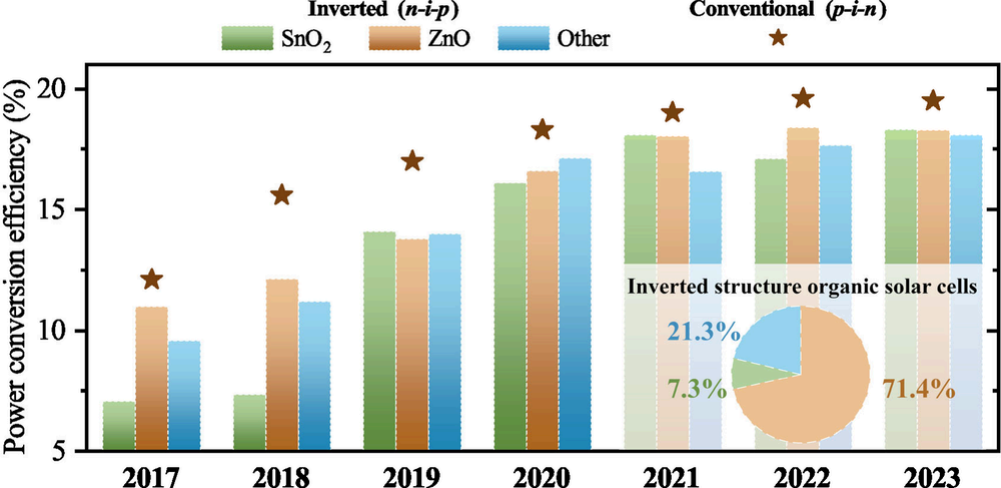
Evolution of reported champion PCEs for single-junction organic solar cells with the two types of device structures. The inset shows the ratio of publications with ZnO, SnO_2_ and other electron transport layers in the 2021–2023 period using “inverted organic solar cells”. Data are from the Web of Science database.

Initially, titanium oxide (TiO_2_) and later zinc oxide (ZnO) served as benchmark electron transport layers (ETLs) in organic photovoltaics. The higher electron mobility and low-temperature processing offered by ZnO triggered this transition.^9,10^ Since then, ZnO has become the preferred option in the field, appearing in 71.4% of the total publications discussing inverted organic solar cells in the time frame between 2021 and 2023. Despite their popularity, reported champion PCEs have not necessarily overcome those with alternative ETLs (Figure 1). Furthermore, ZnO suffers from poor stability under UV light and several evidence have been reported on the fact that it induces photocatalytic degradation of the active layer.^11^

Among the potential alternatives, several n-type polymers such as poly[(9,9-bis(3′-(*N*,*N*-dimethylamino)propyl)-2,7-fluorene)-*alt*-2,7-(9,9-dioctylfluorene)] (PFN) and other organic compounds have been proposed in the past years. However, their low conductivity limits their scalability (layers must be approximately 5 nm), and their cost is still way above those for the metal oxides.^3^

Due to its unique combination of optoelectronic properties and versatile processing, tin oxide (SnO_2_) stands out as a realistic alternative to ZnO. SnO_2_ benefits from high electron mobilities, superior optical transmittance, and lower reactivity to UV light compared to ZnO or TiO_2._^12,13^ Its conduction band is well aligned with the lowest unoccupied molecular orbital (LUMO) of most nonfullerene acceptor molecules employed nowadays (∼4.1 eV), and its deep valence band provides an effective hole-blocking capability.^9^ Its performance as ETL was early proven for dye-sensitized solar cells^14^ and, later on, for perovskite solar cells to a larger extent.^15,16^ Only between 2018 and 2022 were at least 6 review articles on SnO_2_ electron transport layers for perovskite solar cells published,^9,12,15,17−19^ and nowadays, this material is used to reach the highest PCE in the field.^20^ More strikingly, despite its low publication rate in comparison with ZnO, the best efficiency for inverted OSCs in 2023 was obtained using SnO_2._^21^

Within this context, in this Perspective, we discuss if this metal oxide has the potential to become the next benchmark ETL for OSCs. Before doing so, we will review its integration into recent state-of-the-art OSCs, emphasizing one of the most critical factors: its defect chemistry and the strategies employed to achieve efficient and stable OSCs. Importantly, this analysis can inspire future research on inverted OSCs, driving advancements toward large-scale production.

Like other metal oxides, SnO_2_ offers versatile processability with the possibility of depositing thin films from solution processing. The preparation method strongly determines the structural and electronic properties. The most used deposition protocol for the material in OSCs is spin-coating of SnO_2_ nanoparticles (NPs), which typically leads to an amorphous or nanocrystalline structure, depending on the quality of the starting nanoparticles. The first successful synthesis and application of low-temperature NPs-SnO_2_ in OSCs was reported by the Yang Yang group in 2013.^22^ Since then, there has been extensive research to gain control over the NPs properties, increase their colloidal stability, and simplify the synthesis route.^23^ As a result, several NPs-SnO_2_ formulations are commercially available nowadays. Unfortunately, processing SnO_2_ from solution comes along with the challenging control of the material defects and dealing with their serious effects on device performance. This is one of the most critical aspects that currently limits the replacement of ZnO with SnO_2_ in OSCs. Alternative solution-processed methods are sol–gel deposition^23,24^ or chemical bath deposition,^25^ where a higher degree of crystallinity is typically obtained but surface defects are still an issue.

At ambient pressure, the most thermally stable crystal structure of SnO_2_ is the rutile-type tetragonal phase.^26^ Depending on the preparation method, pH, temperature, and other parameters, Sn might be found partially or fully in its most stable oxidation state (Sn^4+^). In some cases, annealing in air is required for complete conversion to stoichiometric form in the bulk. However, it has been shown that SnO_2_ naturally forms nonstochiometric defects on the surface. In some cases, these defects may originate from an incomplete reaction in the synthesis process or the formation of byproducts.^27^ Due to their low formation energy, the dominant crystal defects are tin interstitials (Sn_i_) and oxygen vacancies (V_O_). These mostly form shallow donor states close to the conduction band, which lead to the generation of charge carriers and are responsible for the n-type character of SnO_2_.^28^ However, some of these defect states can also form far from the conduction band minimum, compromising the electron mobility and giving rise to charge-trap states. These are particularly important because they alter the work function and can downgrade the *V*_OC_ and FF of the solar cell.^29,30^ Other typical surface defects that originate at the surface of SnO_2_ are hydroxides. They can exist on the surface of SnO_2_ via binding with either one Sn or two Sn sites, or react with O ions to form water and Sn dangling bonds.^12^ Finally, when NPs-SnO_2_ are employed, the ligands and stabilizers used to maintain the colloidal stability might stay on the surface, introducing another form of impurities that may affect the charge extraction properties.^31^

Overall, the surface defect chemistry of SnO_2_ influences the performance of the solar cell through 1) the formation of surface dipoles with consequent band bending, which can lead to charge accumulation at the interface, 2) the generation of interfacial charge-trap states which can promote nonradiative recombination and 3) modification of its surface free energy with an impact on the active layer morphology e.g. promoting phase segregation (Figure 2b). Moreover, the high reactivity of some surface defects can compromise the device stability.^3,31^

**Figure 2:**
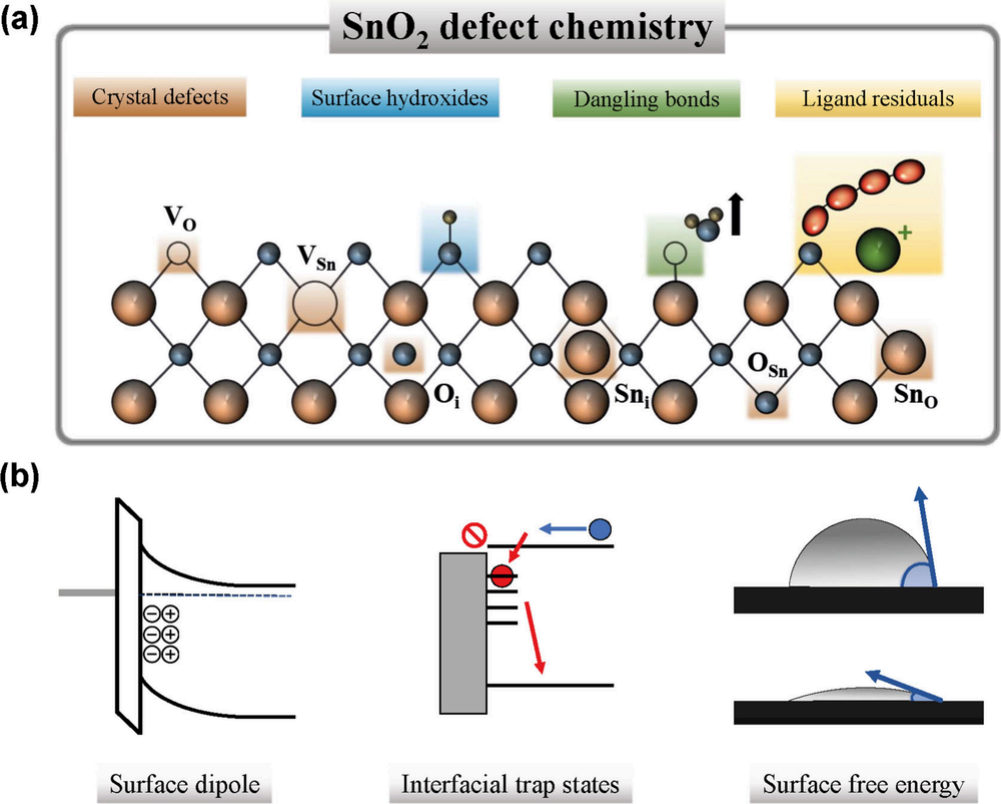
Sketch of (a) defect chemistry of SnO_2_ and (b) main effects on the interface properties in organic solar cells.

Considering the wide defect chemistry of SnO_2_ and the demonstrated variability of the device performance, it is not surprising that this material has not yet become a standard in the field. In particular, the observation of light soaking and/or poor stability in devices with SnO_2_ has hinted at the strong need to combat surface defects.^31,32^

Therefore, to establish SnO_2_ as a high-performing benchmark material for OSCs, it is essential to eliminate or limit the effect of surface defects. In the past few years, several groups have employed different passivation strategies to improve OSCs performance (Table 1).

**Table 1:** Surface Defect Passivation Strategies on SnO_2_ Reported for Organic Solar Cells

**Year**	**Defect**	**Passivation compounds**	**Active layer**	**PCE**_**max**_**[%]**	a**PCE**_**ratio**_	**Device lifetime**	**Ref**
2018	V_O_	Alkali carbonates	PTB7-Th:PC_70_BM	7.35	1.34	90%, 6 w shelf life	(48)
2019	V_O_	Zwitterion sulfobetaine	PTB7-Th:PC_70_BM	8.22	1.84	98%, 4 w shelf life	(33)
2019	–OH	Ethanolamine molecule	PBDB-TF:IT-4F	12.45	1.16		(49)
2022	V_O_	Zwitterion molecule	PM6:Y6	17.12	1.16	36.5%, 1000 h MPP	(34)
2022	V_O_	Anthraquinone molecule	PM6:PB2F:BTP-eC9	18.1	1.16	59.3%, 350 h MPP	(50)
2023	V_O_	NMA molecule	PM6:L8-BO	18.33	1.31	86.6%, 800 h MPP	(21)
2024	–OH and V_O_	Phen-NaDPO molecule	PM6:L8-BO	18.31	1.09	T_80_ = 210 h MPP	(35)
2024	V_O_ and V_Sn_	Urea-PEI	PM6:Y6	16.0	1.18	T_80_ = 260 h MPP	(37)
2024	V_O_	Acrylate oligomer	PM6:Y6-BO	17.0	1.23	77.7%, 450 h MPP	(51)

a PCE_ratio_ is defined as the PCE of the champion passivated device divided by the PCE of the champion reference device.

Notably, zwitterionic small molecules have shown a potential to reduce the oxygen vacancies of SnO_2_.^33,34^ These molecules work as dopants that simultaneously increase the conductivity of SnO_2_ and reduce the surface trap-assisted recombination. In particular, Gao et al. recently employed 4-(dimethyl(pyridine-2-yl) ammonio)butane-1-sulfonate as dopant on SnO_2_ which led to an increase in *V*_OC_ from 0.82 to 0.85 V and an increase in FF from 69.84% to 74. 04%.^34^

More recently, phenanthroline derivatives have been employed as multisite passivation agents on SnO_2_ (Figure 3a,b). Wu et al. used 3-[6-(diphenylphosphinyl)-2-naphthalenyl]-1,10-phenanthroline (Phen-NaDPO) to passivate SnO_2_ surface defects using either a surface treatment^35^ or a self-organization processing strategy.^36^ They demonstrate passivation of Sn dangling bonds through dual coordination which lowers the charge extraction barrier, and demonstrated unprecedented efficiencies in inverted configuration of up to 18.87%.^36^ Interestingly, their evaluation of trap density shows that a narrower distribution of the trap states and even higher performances can be obtained when Phen-NaDPO is incorporated in the active layer solution and is left to self-organize at the SnO_2_ interface. The migration of the passivating agent toward the oxide is explained by its difference in surface free energy with the active layer components, which favor its interaction with SnO_2_.^36^

**Figure 3:**
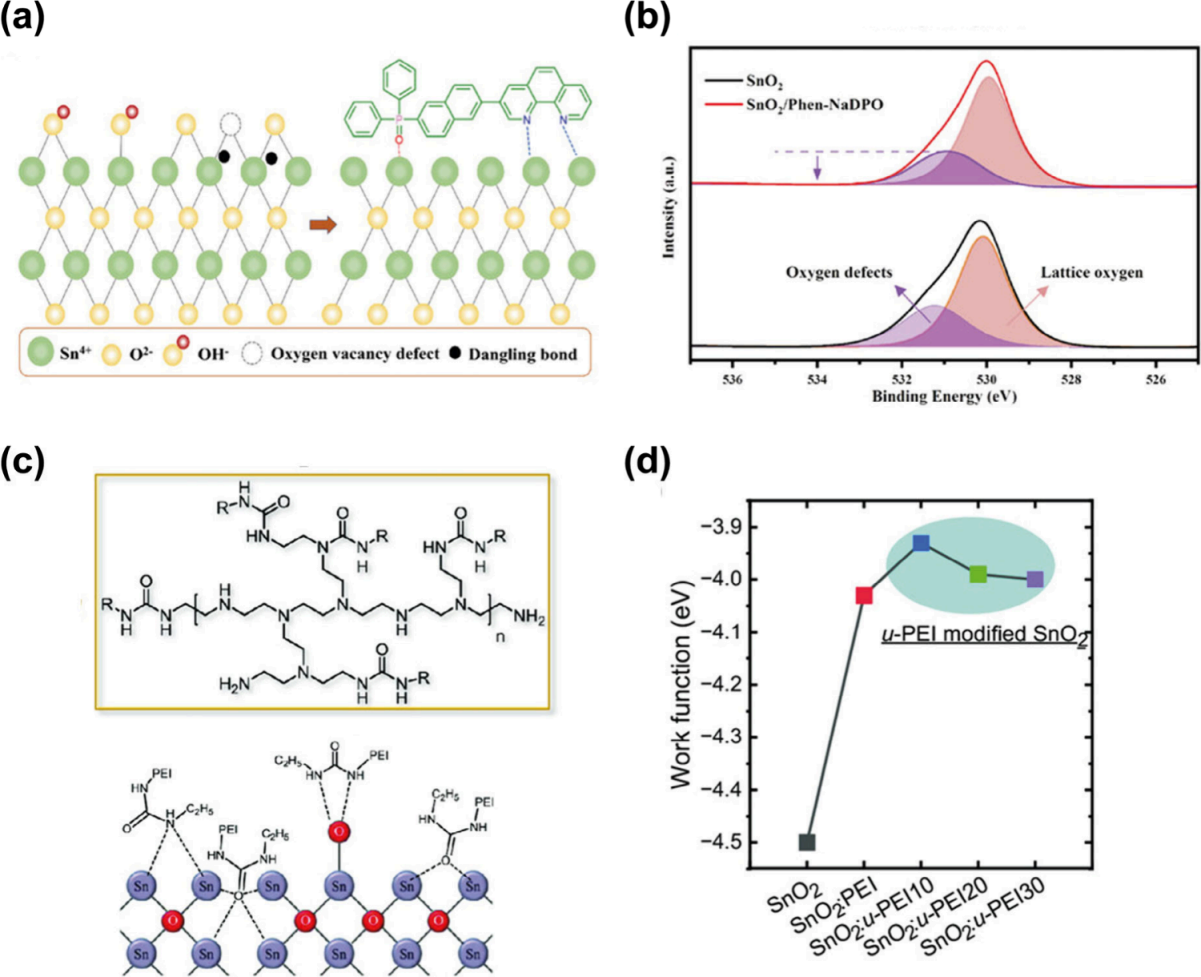
Surface modification of SnO_2_ using molecules with multisite coordination. (a,b) The coordination mechanism of Phen-NaDPO and corresponding XPS showing a drop in the oxygen vacancies. Reproduced with permission from ref (35). Copyright 2023 John Wiley and Sons. (c,d) Coordination mechanism of urea-polyethylenimine and effect of molecule modification on SnO_2_ work function. Reproduced with permission from ref (37). Copyright 2024 John Wiley and Sons.

Finally, the simultaneous passivation of oxygen vacancies and surface hydroxyl groups on SnO_2_ was accomplished by Kim et al. employing a series of urea-functionalized polyethylenimines (Figure 3c). The synergy between the carbonyl and amine terminal groups in the same molecule helps to passivate both types of defects, demonstrating a large increase in the conductivity of the SnO_2_ films. Moreover, its dipole moment reduced the work function of SnO_2_, improving the energy-level matching compared with the control SnO_2_ (Figure 3d).^37^

The arrangement of these coordinating molecules (or passivants) on the surface of SnO_2_ is expected to occur in a disordered manner, which might lead to molecular aggregation on the surface and, in some cases, compromise charge extraction. In contrast, recent advancements in interfacial engineering have highlighted the potential of highly ordered self-assembled monolayers (SAMs), which could address this issue while still benefiting from defect passivation.^38,39^ These SAMs contain anchoring groups, such as phosphonic acid, carboxylic acid, or silane, which chemically react with the oxide surface to form a strong covalent bond, ensuring a well-aligned molecular orientation. Through this process, they can form homogeneous monolayers and adjust the work function by inducing dipoles on the surface.^40^ This strategy has demonstrated promising results with p-type SAMs employed on either the bottom electrode or on top of a metal-oxide layer for organic and perovskite solar cells.^41,42^ Unfortunately, the use of SAMs on the n-side remains underexplored, primarily due to the scarcity of good n-type SAMs.^43^ One of the few successful SAM strategies for OSCs has been to design molecules with electron-withdrawing tail groups that resemble those of the active layer molecules. A remarkable example was reported by Huang et al. and Li et al., where an open azafulleroid SAM was used in solar cells with fullerene-based blends.^44,45^ More recently, Liu et al. proposed several molecules with electron-accepting groups typically found in nonfullerene acceptors (rhodamine, 1,3-indanenione, and 2-(3-ethyl-4-oxothiazolidin-2-ylidene)malononitrile) and successfully applied them to ZnO for high-efficiency OSCs.^46,47^ We speculate that employing n-type SAMs on SnO_2_ will be an effective direction to obtain even higher solar cell performance. Nonetheless, research on the role of surface properties of SnO_2_ (e.g., surface chemistry and roughness) for SAM formation will be imperative for a robust implementation.

Interfaces between the active and the transport layers are one of the most relevant sources of device instability.^52^ Most of the reported passivation strategies displayed in Table 1 positively impacted device lifetimes. A remarkable improvement in both the shelf lifetime and the operational stability was demonstrated by Yu et al., upon passivation of SnO_2_ with an anthraquinone derivative.^50^ The passivated devices kept 97.9% of the original PCE after 2000 h and were stored in dark and inert conditions, in contrast to 82.2% of the control sample. Notably, after 370 h of continuous illumination, the PCE increased from 13.7% to 59.3% of the initial performances. Such improvement was ascribed to a suppressed trap-assisted recombination in the aged devices as a result of the reduction in oxygen vacancies.

Recently, our group has investigated the surface chemistry of the most popular commercially available NPs-SnO_2_ (Alfa-SnO_2_) and found that the residual ligands have a detrimental effect on the stability of the solar cells.^31^ We proposed an easy method to remove the ligands which not only improved the PCE from 15.17% to 16.26% but also led to a more stable >85% of the original PCE after continuous illumination for over 100 h (Figure 4).

**Figure 4:**
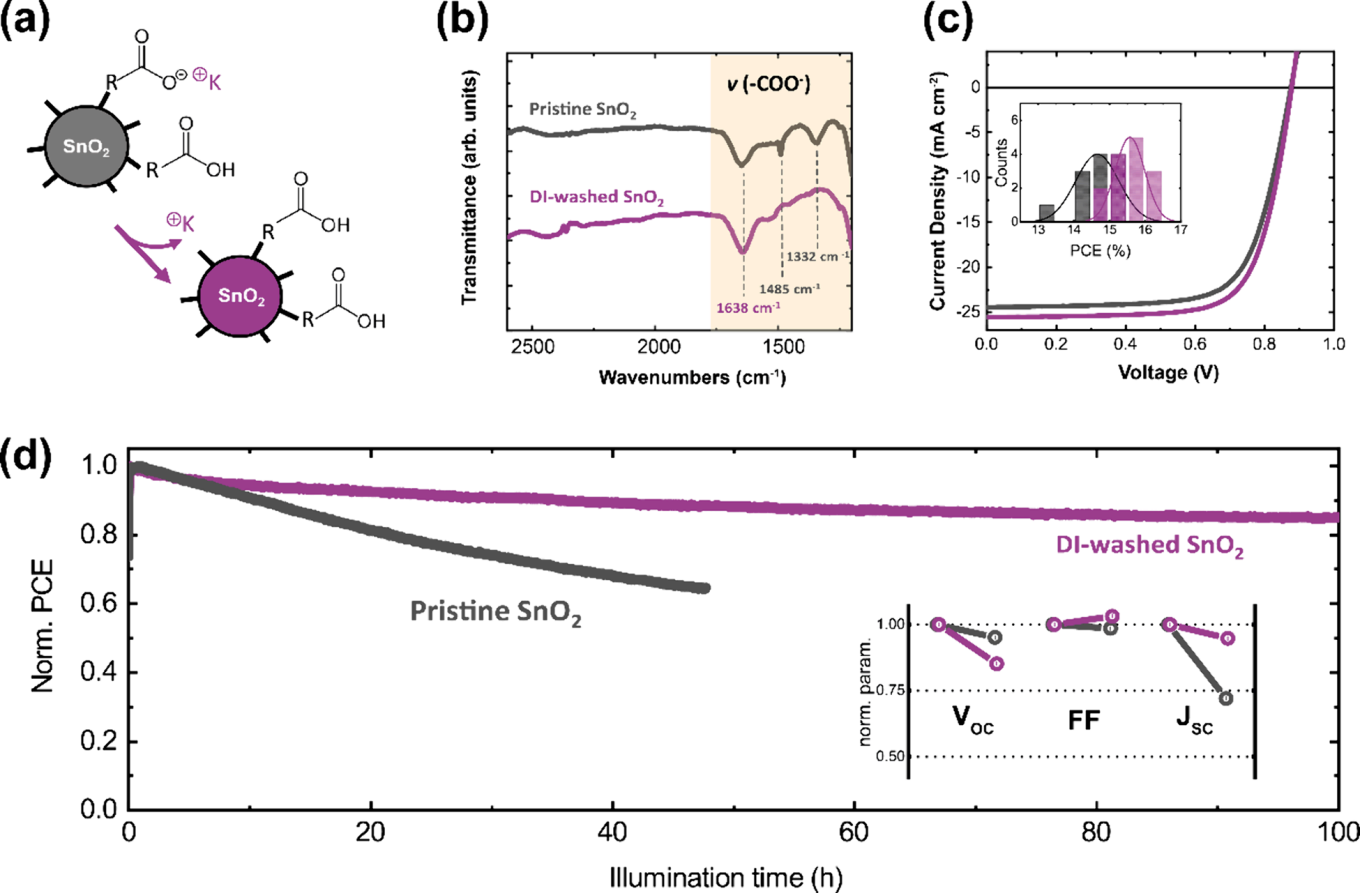
Removal of ligand residuals from SnO_2_ nanoparticles. (a) Schematic of ligand removal. (b) FTIR of pristine and washed SnO_2_. (c) Effect of ligand removal on device performance. (d) Effect of ligand removal on operational stability. Reproduced from ref (31). Copyright 2023 The Authors. Available under CC-BY 4.0.

Here, a few points related to the passivation of SnO_2_ need to be emphasized: whether it is a crystal vacancy, surface hydroxides, dangling bonds, or ligand residuals, it is important to identify which surface defect mostly affects SnO_2_ electron extraction and rationally design the passivating strategy. The employed compounds should be easily accessible or have a simple synthesis, to be easily generalized, and a good compromise between the material quality and scalability of the process should be achieved. A favorable energy alignment with the active layer should be promoted. Finally, the passivation should not compromise the solar cell’s operational stability.

We anticipate that solution-processed SnO_2_ will eventually outperform ZnO, even at larger scales when its surface defects will be controlled. While the morphology control becomes more challenging as one goes from spin-coating to large-area deposition techniques such as slot-die or inkjet printing, some studies have shown promising results with SnO_2_. By using a cosolvent strategy to control its drying rate, Anderson et al. demonstrated the deposition of SnO_2_ layers via slot-die coating and its application in OSCs.^53^ More recently, a PCE of 10.4% was achieved in all-slot-die coated devices using a SnO_2_ layer modified with perylene bisimide.^54^ In another study, Wagner et al. implemented SnO_2_ in a roll-to-roll production line for high-throughput automated optimization.^55^ They showed, by using a 2D combinatorial approach, that the thickness of the printed SnO_2_ and the donor–acceptor ratio are intercorrelated, and ultimately affect the *V*_OC_ losses.

While solution-processed SnO_2_ has great potential to become the benchmark ETL, it is noteworthy that vapor-processed SnO_2_ layers have also been successfully used in OSCs. Their slow growth rate under a controlled vacuum environment ensures conformal high-quality layers, typically with a reduced roughness compared to the solution-processed ones. Atomic layer deposition (ALD) was used by the Riedl group in 2015 to grow SnO_2_ for solar cells with a fullerene-based active layer.^13,56^ They showed that, in contrast to ZnO, ALD-SnO_2_ could offer a constant work function under UV light and maintain a stable *V*_OC_ and FF. Recently, our group has successfully demonstrated that ALD-SnO_2_ can also outperform ZnO in nonfullerene-based OSCs, reaching a PCE of 17.26% and improved operational stability.^57^ Other SnO_2_ vacuum deposition methods include magnetron sputtering, pulsed-laser deposition, e-beam evaporation, and thermal evaporation. Unfortunately, they have been rarely applied in OSCs.^58,59^ While these methods can produce high-quality layers, with good control of the density and thickness, they suffer from low processing speeds and relatively high costs.^9^ Notwithstanding, many surface defects described above for solution-processed SnO_2_ could be avoided by employing vacuum-processed SnO_2_. Other impurities, though, such as unreacted precursor residues have been shown to remain on the surface of ALD-SnO_2_ films when grown at low temperatures (80 °C).^60^ However, the limited number of studies available does not allow us to determine whether the defects in vacuum-processed SnO_2_ are a limiting factor for the performance of state-of-the-art systems. Therefore, there is a need to intensify the investigation of vacuum-based deposition of SnO_2_.

In conclusion, we foresee SnO_2_ becoming the next standard ETL for OSCs provided that its defect chemistry is well addressed. To date, a few small molecules have proven to be effective in passivating SnO_2_ surface defects and improving solar cell performance. Among the best reported so far, multisite coordination molecules have shown efficiencies comparable to those offered by ZnO, the current benchmark ETL. While these advancements are promising, further research is needed to clarify which surface defects have the strongest impact on the performance of OSCs. In this regard, hybrid transport layers where SnO_2_ is functionalized with SAMs will be a key configuration to match the performance of current p-i-n OSCs. Inverse design approaches based on machine learning models, such as the one recently reported for HTL discovery by Wu et al.,^61^ may be helpful in finding new molecules for efficient ETL designs.

Other than efficiency, addressing the stability of SnO_2_-based devices is essential. Even if there is clear evidence that SnO_2_ leads to higher operational and long-term stability than TiO_2_ and ZnO, it can still experience significant degradation under certain conditions, such as high humidity and intense UV exposure.^62^ Therefore, more research is required to unravel the interfacial degradation mechanisms under realistic operating conditions. Finally, vapor deposition methods, such as ALD, should be further explored for OSCs, due to their proven capability to produce high-quality SnO_2_ with fewer surface defects.
